# Loss of SALL1 Promotes Hepatocellular Carcinoma Growth and Is Associated with Poor Clinical Outcome

**DOI:** 10.3390/cancers18091355

**Published:** 2026-04-24

**Authors:** Yoshifumi Saito, Carlos Ichiro Kasano-Camones, Atsumi Tamura, Shioko Kimura, Xiaoting Yu, Yutong Cui, Vorthon Sawaswong, Kristopher W. Krausz, Dong Wang, Aijuan Qu, Yusuke Inoue, Shogo Takahashi, Frank J. Gonzalez

**Affiliations:** 1 Cancer Innovation Laboratory, Center for Cancer Research, National Cancer Institute, National Institutes of Health, Bethesda, MD 20892, USA; 2 Laboratory of Metabolism, Division of Molecular Science, Graduate School of Science and Technology, Gunma University, Kiryu 376-8515, Gunma, Japan; 3 Key Laboratory of Remodeling-Related Cardiovascular Diseases, Department of Physiology and Pathophysiology, School of Basic Medical Sciences, Capital Medical University, Ministry of Education, Beijing 100069, China; 4 Department of General Surgery, Beijing Friendship Hospital, National Clinical Research Center for Digestive Diseases, State Key Laboratory of Digestive Health, Beijing Key Laboratory of Cancer Invasion and Metastasis Research, Capital Medical University, Beijing 100050, China; 5 Center for Food Science and Wellness, Gunma University, Maebashi 371-8510, Gunma, Japan

**Keywords:** SALL1, tumor suppressor, hepatocellular carcinoma, HepG2, Huh-7, xenograft, transcription factor

## Abstract

Background/Objectives: Hepatocellular carcinoma (HCC) remains a major malignancy with high incidence and mortality, in part due to its diverse etiology and intratumoral heterogeneity, which contributes to drug resistance and frequent recurrence. SALL1 (Spalt-Like Transcription Factor 1), a zinc-finger transcription factor, was reported to function as a tumor suppressor in several cancers, including breast cancer and glioma, and accumulating evidence support its involvement in tumor biology. In this study, the role of SALL1 in HCC was examined. Methods: Public RNA and protein databases derived from human HCC were interrogated. Western blotting quantification of clinical HCC for SALL1 levels was carried out. Cell culture and xenograft studies were performed using genetically modified HCC tumor cells. Results: As revealed by pubic RNA and protein database analysis and further western blotting quantification of clinical samples of HCC, SALL1 is decreased in human HCC. The effect of reduced SALL1 expression on the tumorigenic properties and transcriptional regulation in HCC was then examined. Knockdown of SALL1 in the HCC cell lines Huh7 and Hep3B, enhanced cell proliferation in vitro and accelerated tumor growth in a xenograft mouse model, suggesting that lower SALL1 expression increases cell proliferation and tumorigenesis in HCC. RNA-seq and ChIP analyses further identified three novel candidate target genes (*SLC6A14*, *GABRG1*, and *AKR1B10*), suggesting that SALL1 may exert a tumor-suppressive effect, at least in part, through negative regulation of these genes. Conclusions: These findings establish SALL1 as a possible tumor suppressor and provide new insights into the biological significance of SALL1 downregulation in HCC. SALL1 could be a candidate prognostic marker and a potential therapeutic target.

## Simple Summary

Liver cancer is a major cause of cancer-related deaths worldwide, and better treatment strategies are still needed because many patients are not eligible for curative treatment or develop recurrence after therapy. In this study, SALL1, a protein involved in the regulation of gene expression, was markedly reduced in liver cancer cells. Forced reduction in SALL1 expression in liver cancer cells promoted cell growth and tumor progression. These findings suggest that lower SALL1 expression may be associated with increased liver cancer progression. This study enhances our understanding of the molecular circuits involved in liver cancer and suggests that SALL1 may be a potential therapeutic target in this disease.

## 1. Introduction

Hepatocellular carcinoma (HCC) is the sixth most commonly diagnosed cancer and the second leading cause of cancer-related deaths worldwide [1,2,3]. Although curative treatment by surgical resection or liver transplantation is possible for some patients, only about 30% are eligible, and recurrence is frequent [4]. While molecular targeted therapies and immunotherapies have improved outcomes, predictive biomarkers remain limited and treatment responses are often heterogeneous [5]. The diverse etiological backgrounds and marked molecular heterogeneity of HCC further contribute to therapeutic resistance, underscoring the need for new treatment strategies [6,7,8,9].

Transcription factors (TFs) are essential regulators of biological homeostasis, as they recognize specific DNA sequences, bind to them, and control downstream target gene expression, thereby orchestrating a variety of cellular processes [10]. TFs broadly govern critical cancer-related processes, including proliferation, migration, angiogenesis, apoptosis, signal transduction, and senescence. Certain transcription factors, such as MYC and YAP/TAZ, were identified as major oncogenic regulators that play central roles in HCC as well as in other cancers [11,12]. In contrast, some liver-enriched transcription factors, including HNF4α and HNF1α, were reported to exert tumor-suppressive functions in HCC [13]. These findings indicate that TFs can function in either tumor-promoting or tumor-suppressive manners depending on the biological context, and that clarifying the role of individual TFs may provide important insights into HCC progression and potential therapeutic strategies.

The human SALL gene family (SALL1-SALL4) comprises zinc finger transcription factors involved in development, differentiation, and epigenetic regulation [14,15,16,17,18,19,20,21,22,23]. Among these family members, SALL4 is known to function as an oncogene in various cancers, whereas SALL2 acts as a tumor suppressor [24]. SALL1 functions as a transcriptional repressor through interaction with the NuRD complex and has been implicated in stemness and chromatin regulation [18,19,22,25,26,27]. Recent studies have shown that SALL1 can act as either a tumor suppressor or an oncogene in a cancer-type-dependent manner, functioning as a tumor suppressor in breast cancer and glioma, but as an oncogene in acute myeloid leukemia and colorectal cancer [28,29,30,31,32,33]. However, the role of SALL1 in HCC remains poorly understood.

Previous studies showed that overexpression of SALL1 in HCC cell lines suppressed cell proliferation, migration and invasion, supporting a tumor suppressive role in HCC [34]. However, it remains unclear whether and how SALL1 loss promotes proliferative capacity and tumor progression in HCC. In this study, gene expression analysis using the TCGA database and human samples revealed that *SALL1* mRNA expression is downregulated in HCC tissues, and protein-level data also demonstrated a significant reduction in SALL1 protein expression in HCC. Furthermore, SALL1 knockdown in HCC cell lines increased cell proliferation and tumor growth. Taken together, these results suggested that downregulation of SALL1 in HCC may contribute to tumor progression. These findings also highlight the potential relevance of SALL1-related pathways as a therapeutic consideration in HCC.

## 2. Materials and Methods

### 2.1. Animals

All animal experiments were performed in accordance with the Institute of Laboratory Animal Care international guidelines and approved by the National Cancer Institute Animal Care and Use Committee (protocol number: LM-092-3-AA). Immunodeficient NOD Cg-*Prkdc^scid^Il2rg^tm1Wjl^*/SzJ (NSG) mice (Jackson Laboratory, Bar Harbor, ME, USA) were used in this study. The mice were maintained in sterile cages and given sterile regular diet and drinking water supplemented with sulfadiazine and trimethoprim to prevent infection. Animals were kept in a pathogen-free animal facility under a standard 12 h light/dark cycle (lights on at 7 AM). Both male and female mice aged 6 to 8 weeks were included in the experiments.

### 2.2. Human Samples

All human samples were collected from Beijing Friendship Hospital, Capital Medical University, under strict adherence to ethical guidelines. Written informed consent was obtained from all participants before collection. All the procedures were approved by the Medical Ethics Committee of Beijing Friendship Hospital (ethics identification number: 2023-P2-145-02). Human non-viral-HCC tumor tissues, adjacent liver specimens and paraffin-embedded sections were obtained at the Beijing Friendship Hospital, Capital Medical University. The characteristics of patients are listed in Supplementary Table S1.

### 2.3. Xenograft Mouse Model

Cell transplantation into NSG mice was performed using Matrigel (Corning Incorporated, Corning, NY, USA; Cat. No. 354234). A total of 1 × 10^6^ cells (Huh7 and Hep3B cells) per mouse were prepared by mixing equal volumes (50 μL each) of DMEM containing 4.5 g/L glucose, L-glutamine, and sodium pyruvate (Corning Incorporated, Corning, NY, USA; Cat. No. 10-013-CV) and Matrigel (1:1 ratio). In total, 100 μL of the Matrigel–cell mixture was injected subcutaneously into the right flank of each mouse. Tumor size was measured weekly using calipers to determine the length and width. Tumors were excised from all groups when the longest tumor diameter reached approximately 2 cm, which was defined as the experimental endpoint by experimental guidelines.

### 2.4. Cell Culture

HepG2, Hep3B, PLC/PRF/5 and SK-Hep-1 cells were obtained from the American Type Culture Collection (ATCC) (Manassas, VA, USA). 293FT cells were purchased from Thermo-Fisher Scientific (Waltham, MA, USA), and Huh7 cells were provided by the Laboratory of Human Carcinogenesis, National Cancer Institute (Bethesda, MD, USA). All cell lines were maintained in DMEM containing 4.5 g/L glucose, L-glutamine, and sodium pyruvate (Corning Incorporated, Corning, NY, USA; Cat. No. 10-013-CV), supplemented with 10% FBS (Gemini Bio-Products, West Sacramento, CA, USA; Cat No. 100-106-500) and 1% antibiotics (Gibco, Thermo Fisher Scientific, Waltham, MA, USA; Cat. No. 15240-062). Cells were cultured at 37 °C in a humidified incubator with 5% CO_2_. For routine passage, cells were rinsed with PBS (Corning Incorporated, Corning, NY, USA; Cat. No. 21-040-CV) and detached using 0.25% trypsin-EDTA (1×) (Gibco, Thermo Fisher Scientific, Waltham, MA, USA; Cat. No. 25200-056).

### 2.5. mRNA Quantification

Total mRNA was isolated from cultured cells and tumor tissues using TRIzol Reagent (Invitrogen, Thermo Fisher Scientific, Waltham, MA, USA; Cat. No. 15596018). Following lysis, RNA was separated by chloroform extraction (Macron Fine Chemicals, Avantor, Radnor, PA, USA; Cat. No. 4440-24), precipitated with isopropyl alcohol (Aqua Solutions, Deer Park, TX, USA; Cat. No. 67-63-0) and washed with 75% ethanol. Samples were centrifuged for 15 min at 12,000× rpm, 4 °C. RNA concentration was determined using a NanoDrop spectrophotometer. For reverse transcription, 500 ng of total RNA from each sample was used with qScript Reverse Transcriptase (Quantabio, Beverly, MA, USA; Cat. No. 84003). RT-qPCR was carried out using the PerfeCTa SYBR Green Supermix (Quantabio, Beverly, MA, USA; Cat. No. 84073) on a QuanStudio 7 Flex System (Thermo-Fisher Scientific, Waltham, MA, USA). Primer sequences used for mRNA quantification are provided in Supplementary Table S2.

### 2.6. Western Blots

Proteins were extracted in RIPA buffer (Sigma-Aldrich, St. Louis, MO, USA; Cat. No. 20-188) supplemented with a protease and phosphatase inhibitor cocktail (Thermo Fisher Scientific, Waltham, MA, USA; Cat. No. 1861284). After incubation on ice for 20 min, lysates were centrifuged at 14,000 × rpm for 5 min at 4 °C, and supernatants were transferred to fresh microcentrifuge tubes. Protein concentration was measured using the Pierce BCA Protein assay kit (Thermo Fisher Scientific, Waltham, MA, USA; Cat. No. A65453), by reading absorbance at 562 nm according to the manufacturer’s instructions. Protein lysates were mixed with 4× Laemmli Sample Buffer (Bio-Rad Laboratories, Hercules, CA, USA; Cat. No. 1610747) and heated at 95 °C for 5 min. Equal amounts of protein (60 μg per lane) were resolved by SDS-PAGE using Criterion TGX precast gels (Bio-Rad Laboratories, Hercules, CA, USA; Cat. No. 5671085) in Tris/Glycine/SDS buffer (Bio-Rad Laboratories, Hercules, CA, USA; Cat. No. 1610732) at 140 V for approximately 1 h. Proteins were then transferred onto PVDF membrane (0.2 μm, Trans-Blot Turbo Transfer Pack Midi Format: Bio-Rad Laboratories, Hercules, CA, USA; Cat. No. 1704157) using the Trans-Blot Turbo system (Bio-Rad Laboratories, Hercules, CA, USA). Membranes were blocked with 5% BSA in Tris-buffered saline with Tween-20 (TBST) at room temperature for 1 h and incubated with primary antibodies in TBST for 1 h at room temperature. Anti-SALL1 (Abcam, Cambridge, MA, USA; Cat. No. ab31526), β-actin (Cell Signaling Technology, Danvers, MA, USA; Cat. No. 8457S) and GAPDH (Cell Signaling Technology, Danvers, MA, USA; Cat. No. 5174S) antibodies were each used at a dilution of 1:1000 in 5% BSA. After primary antibody incubation, membranes were washed three times for 10 min each with TBS (KD Medical, Columbia, MD, USA; Cat. No. RGE-3385) containing 0.1% Tween-20 (Sigma-Aldrich, St. Louis, MO, USA; Cat. No. P2287). HRP-conjugated anti-rabbit IgG secondary antibody (Cell Signaling Technology: #7074) was diluted 1:10,000 in 5% BSA and incubated with membranes for 45 min at room temperature. Membranes were subsequently washed three times for 10 min each with TBS containing 0.1% Tween-20, followed by a final 5 min wash with TBS. Signals were detected using the SuperSignal West Dura Extended Duration Substrate (Thermo Fisher Scientific, Waltham, MA, USA; Cat. No. 34076) and imaged with a ChemiDoc MP Imaging System (Bio-Rad Laboratories, Hercules, CA, USA). Band intensities were quantified using Image Lab software (Bio-Rad Laboratories, Hercules, CA, USA) and normalized to β-actin (ACTB) as an internal control. For total protein visualization, whole gels loaded with 30 μg of protein per lane were stained after SDS-PAGE with Coomassie Brilliant Blue staining solution (0.1% *w*/*v*, methanol: 45% *v*/*v*, acetic acid: 10%) (Bio-Rad Laboratories, Hercules, CA, USA; Cat. No. 161-0406). Gels were destained for several hours in solution containing 45% methanol and 10% acetic acid (Sigma-Aldrich, St. Louis, MO, USA; Cat. No. 64-19-7), and protein levels in all lanes were assessed visually.

### 2.7. Virus Packaging and Infection

Lentiviral vectors and packaging plasmids were purchased from VectorBuilder Inc. (Chicago, IL, USA; Cat. No. VB221011-1423fph and VB010000-0013dtn) and Addgene (Watertown, MA, USA; Cat. No. 12259 and 12260). Plasmids were amplified by culturing plasmid-harboring *E. coli* in LB medium (Research Products International Corp., Mt. Prospect, IL, USA; Cat. No. L24045-5000.0) containing carbenicillin (Sigma-Aldrich, St. Louis, MO, USA; Cat. No. C1389) at 37 °C for 16 h, and purified using a Maxi prep kit (Qiagen, Germantown, MD, USA; Cat. No. 12965). For lentiviral packaging, 293FT cells were used. Cells at 80–90% confluency in a 15 cm dish (Corning Incorporated, Corning, NY, USA; Cat. No. 430599) were incubated with chloroquine (1:10,000, Sigma-Aldrich, St. Louis, MO, USA; Cat. No. C6628-25G) for 4 h at 37 °C. Transfection was performed using polyethylenimine (PEI; Polyscience, Niles, IL, USA; Cat. No. 23966-1) with three plasmids—psPAX2, pMD2.G, and the transfer vector—mixed with 0.9% saline (KD Medical, Columbia, MD, USA; Cat. No. RGC-3290) and incubated for 20 min before addition to the cells. Cells were maintained in medium containing 10% FBS without antibiotics. At 24 h post-transfection, the medium was replaced with 10% FBS and 1% antibiotics, and after an additional 24 h, the culture medium was collected. The supernatant was centrifuged at 6000× *g* for 15 min at 4 °C to remove cell debris, followed by centrifugation at 6000× *g* for 2 h at 4 °C. The pellet was resuspended in DPBS (Gibco, Thermo Fisher Scientific, Waltham, MA, USA; Cat. No. 14190144) to obtain the lentiviral stock. For infection, cells were seeded at 1 × 10^5^ cells/well in 12-well plates (Corning Incorporated, Corning, NY, USA; Cat. No. 3515). Polybrene supplied with lentiviral preparation (1:1000; VectorBuilder Inc., Chicago, IL, USA) was added to the culture medium, and the lentiviral solution was added at a multiplicity of infection (MOI) of 2. The shRNA sequences used in this study are listed in Supplementary Table S3.

### 2.8. Cell Proliferation

Cell proliferation was assessed using the CCK-8 assay (DOJINDO Molecular Technologies, Rockville, MD, USA; Cat. No. CK04-13). Cells were seeded at 2.5 × 10^3^ cells/well into 96-well plates (Corning Incorporated, Corning, NY, USA; Cat. No. 3596). Absorbance at 450 nm was measured every 24 h, and culture medium was replaced every 48 h.

### 2.9. 3D Culture

3D cell culture was performed using Matrigel (Corning Incorporated, Corning, NY, USA; Cat. No. 354234). Matrigel was mixed with the cell suspension (1 × 10^3^ cells) at a 1:1 ratio (50 μL each), and the 100 μL mixture was carefully added to the center of each well of 12-well plate (Corning Incorporated, Corning, NY, USA, Cat. No. 3515). The Matrigel–cell mixture was incubated at 37 °C for 15 min to allow gelation, followed by the addition of 1 mL DMEM supplemented with 10% FBS and 1% antibiotics to each well. Culture was maintained for 14 days with medium changes every 72 h. Cell viability was assessed using a CCK-8 assay. After adding CCK-8 reagent, cells were incubated at 37 °C for 4 h and absorbance was measured at 450 nm.

### 2.10. RNA Sequencing

RNA sequencing (RNA-seq) was performed using Huh7 stably expressing shRNA against SALL1, or SALL1 expression vector. An RNeasy Plus Kit (Qiagen, Germantown, MD, USA; Cat. No. 74136) was used to extract and purify total RNA from cells. Cells were cultured using 6-well plates for 48 h and were harvested following the protocol supplied with the RNeasy Plus Kit. The purity and concentration were verified using the Tapestation system by the CCR Genomics Core Facility. Library preparation and RNA sequencing were carried out by the National Cancer Institute Sequencing Facility. The Illumina libraries were prepared using the Total RNA Ligation and Ribo Zero plus kit (Illumina, San Diego, CA, USA). Libraries were pooled together and sequenced on a NextSeq 2000 P2 flow cell (Illumina, San Diego, CA, USA) as a 2 × 101 bp pair-end run. Demultiplexing was performed using the Illumina Bcl2fastq v 2.20 software tool. The sequencing reads were trimmed by adapters and low-quality bases using Cutadapt (v1.18). The trimmed reads were mapped to the human reference genome (hg38) and the annotated transcripts (GENCODE v30) using STAR aligner (v2.7.0f) with the two-pass alignment option. RSEM (version 1.3.1) was used for gene and transcript quantification based on the GENCODE annotation file. Raw count data generated by the National Cancer Institute Sequencing Facility was analyzed using the iDEP platform (https://bioinformatics.sdstate.edu/idep96/, accessed on 6 April 2026) [35]. An FDR < 0.05 compared to control was used for multiple testing and to limit false positives, and an FC ≥ 2 was used to focus on genes with biologically meaningful effect size. Potential batch effects were assessed by principal component analysis (PCA) of normalized RNA-seq expression data. RNA-seq was performed using three independent biological replicates per condition.

### 2.11. Chromatin Immunoprecipitation

Chromatin immunoprecipitation (ChIP) assays were carried out using a SimpleChIP Plus Enzymatic Chromatin IP Kit (Magnetic Beads) (Cell Signaling Technology, Danvers, MA, USA; Cat. No. 9005) in accordance with the manufacturer’s instructions. For ChIP-qPCR analysis, Huh7 cells stably expressing shRNA against *SALL1* mRNA or stably expressing control shRNA were seeded in 15 cm dishes at a density of 4 × 10^6^ cells/dish. For crosslinking, formaldehyde was added directly to 20 mL of culture medium to a final concentration of 1%, and cells were incubated at room temperature for 10 min. Isolated nuclear fractions were then treated with micrococcal nuclease at 37 °C for 20 min to digest chromatin. Nuclear membranes were disrupted using an Ultrasonic Processor (Cole-Parmer, Vernon Hills, IL, USA) with 15 cycles of 1 s sonication at 20% amplitude, each separated by 5 s intervals. Immunoprecipitation was performed in triplicate using anti-SALL1 antibody (Abcam, Cambridge, MA, USA; Cat. No. ab41974), with normal mouse IgG antibody (Cell Signaling Technology, Danvers, MA, USA; Cat. No. #68860) used as the control IgG. Antibody–chromatin complexes were incubated overnight at 4 °C and then captured with protein G magnetic beads for 2 h at 4 °C. The precipitated chromatin was treated with proteinase K for 2 h at 65 °C, and DNA was purified using a spin column. ChIP-qPCR was performed using the PerfeCTa SYBR Green Supermix (Quantabio, Beverly, MA, USA; Cat. No. 95054-500) in the QuanStudio 7 Flex System. Enrichment (Thermo Fisher Scientific, Waltham, MA, USA) was calculated using the % input method. Primer sequences used for ChIP-qPCR are listed in Supplementary Table S4.

### 2.12. Dataset Analysis

mRNA levels were analyzed using the UCSC Xena platform (https://xenabrowser.net/, accessed on 6 April 2026) [36]. Protein levels and correlation were analyzed using the UALCAN platform (https://ualcan.path.uab.edu/index.html, accessed on 6 April 2026) [37]. TCGA dataset was used for mRNA analysis and the CPTAC dataset was employed for protein level analysis. mRNA expression levels across histological grades (grades 1–4) were analyzed using the UALCAN platform. Survival analysis was performed using the Human Protein Atlas (HPA) (https://www.proteinatlas.org/, accessed on 6 April 2026) [38].

### 2.13. scRNA-Seq Analysis

Raw count matrices and metadata of publicly available datasets were obtained from the GEO (accession number: GSE242889) [39]. Gene–cell count tables were imported into R (v4.3.1) and processed using the Seurat package (v5.0). A Seurat object was created from the raw counts and corresponding metadata. Cells were annotated based on the provided cell type and patient information. The dataset was split by sample origin, and each subset was independently normalized using Seurat’s *LogNormalize* method. Highly variable genes (n = 2000) were identified per sample using the *vst* method. Shared variable features across samples were selected with SelectIntegrationFeatures, and anchors were computed using FindIntegrationAnchors. Data integration was performed with IntegrateData (dims = 1–30), followed by scaling, principal component analysis (PCA, 50 components), and dimensionality reduction with t-distributed stochastic neighbor embedding (t-SNE). Clustering was carried out using a shared nearest-neighbor graph constructed from the top 30 principal components, followed by Louvain clustering. Cluster-specific marker genes were identified using FindAllMarkers in Seurat with the following criteria: genes expressed in at least 25% of cells per cluster (min.pct = 0.25), log2 fold change ≥ 0.25, and positive differential expression only. For hepatocytes, average SALL1 expression per patient per site (normal vs. tumor) was determined. Statistical comparison between conditions was performed on patient-level means using the Wilcoxon signed-rank test and paired *t*-test.

### 2.14. Statistical Analysis

Sample sizes are presented in the figure legends. Statistical analysis was performed using GraphPad Prism (GraphPad Software Version 10.6.1). Experimental values represent mean ± SEM. Statistical significance between 2 groups was determined using 2-tailed Student’s *t* test. For analyses using publicly available datasets, statistical methods and sample sizes were defined by the respective databases. Unless otherwise stated, n represents the number of independent biological replicates. *p* values were calculated with confidence intervals of 95%. A *p* value less than 0.05 was considered statistically significant.

## 3. Results

### 3.1. SALL1 Is Downregulated at the mRNA and Protein Level in HCC

**Figure 1:**
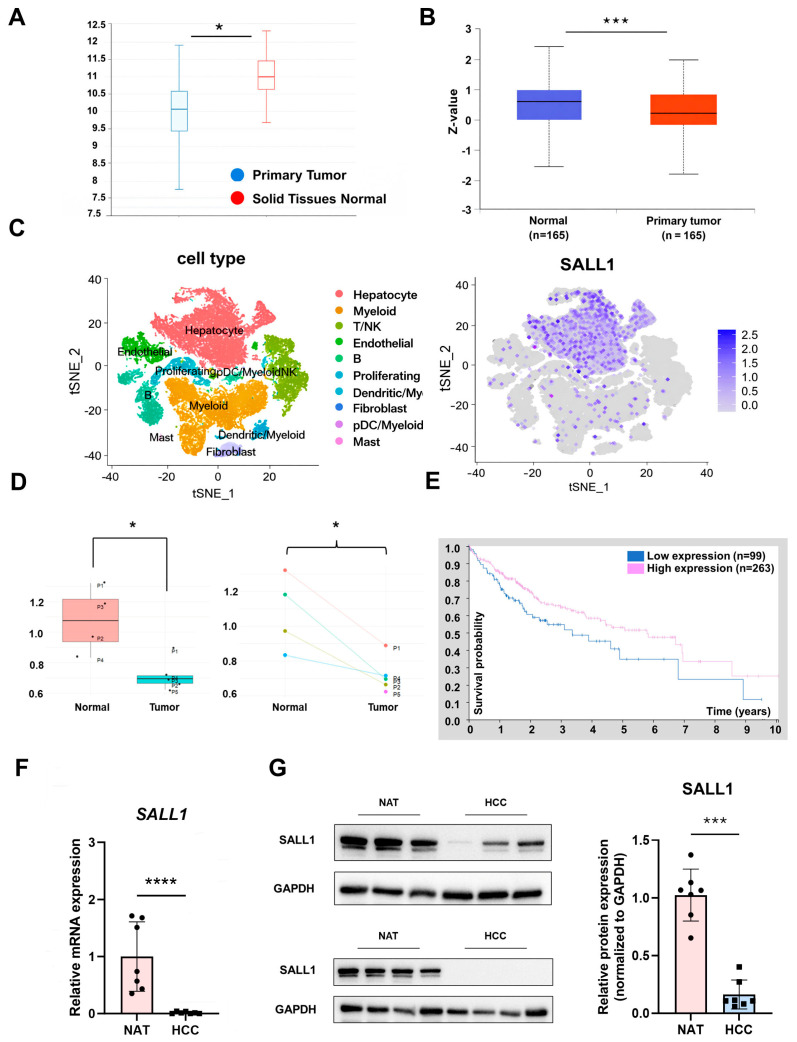
**SALL1 is downregulated at the mRNA and protein levels in HCC.** (**A**) *SALL1* mRNA levels in TCGA-LIHC (normal solid tissues vs. primary tumor) assessed using the UCSC Xena platform. The y-axis indicates expression level. (Normal: n = 50, primary tumor: n = 438). (**B**) SALL1 protein levels in TCGA-LIHC (normal vs. primary tumor) assessed using the UALCAN platform. (Normal: n = 165, primary tumor: n = 165). The y-axis indicates Z-scores, which represent the number of standard deviations from the mean expression level in the dataset. (**C**) Integrated analysis of a public human HCC single-cell RNA-seq dataset containing tumor tissues and adjacent normal liver tissues. Cells from both tissue types were combined and clustered according to cell-type-specific transcriptional profiles (left), and SALL1 expression was mapped across the identified cell clusters to determine its cellular localization (right). (**D**) SALL1 expression in a public human HCC single-cell RNA-seq dataset was further compared between adjacent normal liver and HCC tissues using SALL1-positive cells in panel (**C**). Box-and-whisker plot showing SALL1 expression levels in normal and tumor tissues (left); paired patient-matched comparison of SALL1 expression between adjacent normal and tumor tissues from individual patients (P1–P5) (right). Dots represent individual patients. (**E**) Kaplan–Meier survival curves for patients stratified by SALL1 expression (blue: SALL1-low expression; red: SALL1-high expression). Patients were stratified based on median SALL1 expression. Tick marks indicate censoring. (Low expression, n = 99; high expression, n = 263.) Log-rank test, *p* = 0.029. (**F**) Expression analysis of *SALL1* mRNA levels using human samples subjected to RT-qPCR (NAT: n = 4, HCC: n = 8). NAT: Normal Adjacent Tissue. (**G**) Protein expression analysis of SALL1 in human samples analyzed by Western blotting (n = 7). Band intensities were quantified and normalized to GAPDH. Data are presented as mean ± SEM. Statistical significance was determined using two-tailed Student’s *t*-test, Wilcoxon signed-rank test and paired *t*-test for RT-qPCR, Western blotting, and scRNA-seq analysis. For analyses using the UCSC Xena, UALCAN, and Human Protein Atlas (HPA) platforms, statistical analyses were performed automatically within each platform. * *p* < 0.05, ** *p* < 0.01, *** *p* < 0.001, **** *p* < 0.0001 compared with each control group.

Previous studies reported that SALL1 overexpression suppresses cell proliferation, invasion, and migration in HCC cell lines, supporting a potential tumor-suppressive role for SALL1 [34]. However, the expression level of SALL1 in HCC patients and the functional impact of SALL1 downregulation in HCC progression remained unclear. To investigate whether SALL1 expression is altered in HCC patients, SALL1 expression was explored in the TCGA liver hepatocellular carcinoma (TCGA-LIHC) cohort using the UCSC Xena platform. Analysis of TCGA RNA-seq expression data revealed that SALL1 was significantly decreased in tumors compared with normal tissues in the overall cohort (Figure 1A). In addition to mRNA expression, SALL1 was also significantly decreased at the protein level in HCC tissues, as revealed by UALCAN proteomic analysis (Figure 1B). UALCAN was also used to analyze *SALL1* mRNA expression across histological grades (grade 1–4) (Supplementary Figure S1A). A significant reduction in *SALL1* mRNA was observed in grade 3 (poorly differentiated) tumors, whereas grade 1 (well differentiated) and 2 (moderately differentiated) showed a decreasing trend without statistical significance. Grade 4 (undifferentiated) tumors also exhibited a trend toward lower *SALL1* mRNA without statistical significance which could not be confirmed due to the limited sample size. Subsequent expression analysis using publicly available scRNA-seq datasets revealed that *SALL1* mRNA is predominantly enriched in hepatocytes, the primary site of liver-specific functions (Figure 1C). Moreover, expression analysis of SALL1 comparing normal and HCC tissues from five HCC patients revealed that SALL1 was significantly reduced in HCC tissues (Figure 1D). Taken together with the results from database-based expression analyses, these findings suggest that downregulation of SALL1 may be associated with poor prognosis in HCC. Therefore, survival analysis was performed using Kaplan–Meier survival curves. Survival analysis using the Human Protein Atlas showed that patients with low SALL1 expression had significantly worse prognosis than those with high SALL1 expression (Figure 1E). In addition to expression and survival analyses using public datasets, SALL1 expression was examined in human samples. *SALL1* mRNA and SALL1 protein was significantly decreased in HCC tissues (Figure 1F,G). The SALL1 expression pattern across several HCC cell lines was also examined, revealing consistent results with previous reports: high expression in well-differentiated cell lines such as HepG2, Huh7 and Hep3B, but low expression in poorly differentiated cell-lines such as PLC/PRF/5 and SK-Hep-1 (Supplementary Figure S1B,C). Given that the HepG2 cell line was derived from a hepatoblastoma, rather than adult HCC, Huh7 and Hep3B were selected for further analysis using stably expressed shRNA against SALL1 for knockdown.

### 3.2. Loss of SALL1 Expression Enhances Proliferative Phenotypes in HCC Cell Lines

**Figure 2:**
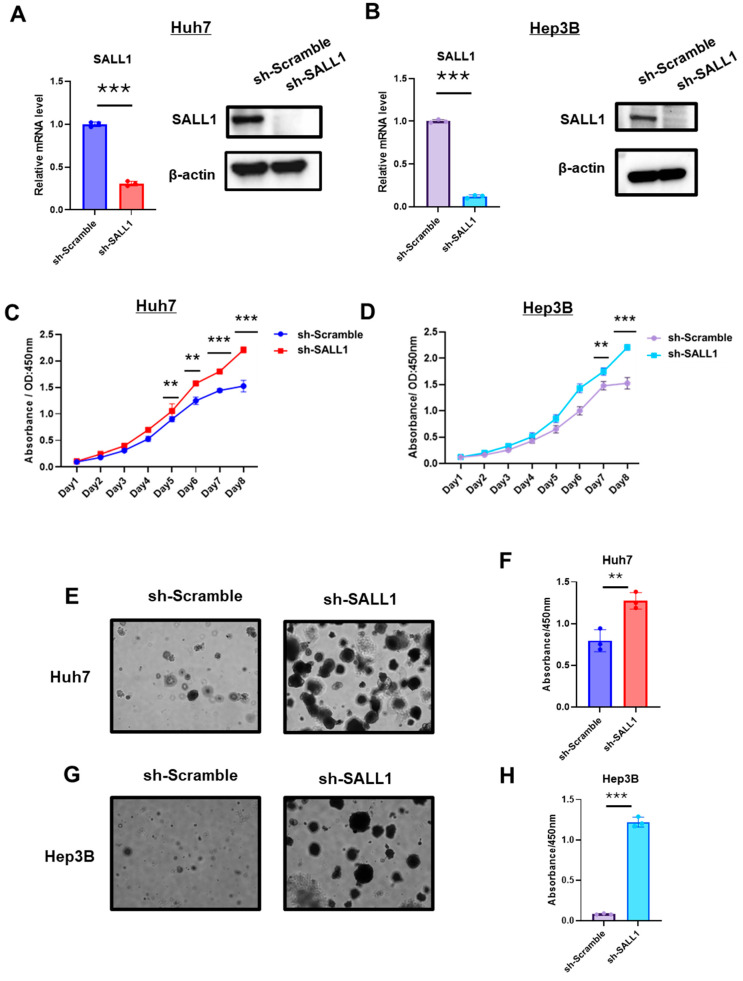
**Loss of SALL1 expression drives aggressive phenotypes in HCC cell lines.** (**A**,**B**) Validation of SALL1 knockdown in Huh7 (**A**) and Hep3B (**B**) cells. *SALL1* mRNA expression was measured by RT-qPCR (left), and SALL1 protein expression was evaluated by Western blotting (right) in cells transduced with sh-Scramble or sh-SALL1. (**C**,**D**) Effect of SALL1 knockdown on cell proliferation under conventional 2D culture conditions in Huh7 (**C**) and Hep3B (**D**) cells. (**E**,**G**) Representative images of 3D Matrigel culture-based proliferation assays in Huh7 (**E**) and Hep3B (**G**) cells, showing increased spheroid growth/proliferative activity in SALL1-knockdown cells compared with sh-Scramble control. Control cells (sh-Scramble) are shown on the left, and SALL1-knockdown cells (sh-SALL1) are shown on the right. (**F**,**H**) Quantification of proliferative capacity in 3D culture by CCK-8 assay in Huh7 (**F**) and Hep3B (**H**) cells. All experiments were performed using three biological replicates for each group (n = 3 per group). Data are presented as mean ± SEM. Statistical significance was determined using two-tailed Student’s *t*-test. ** *p* < 0.01, *** *p* < 0.001 compared with sh-Scramble group (control).

To investigate whether reduced expression of SALL1 affects the phenotype of HCC, well-differentiated HCC cell lines expressing shRNA against SALL1 were established (Figure 2A,B). Cell proliferation assays revealed a significant increase in cell proliferation rates in both Huh7 and Hep3B cell lines lacking SALL1 (Figure 2C,D). In Matrigel-based 3D cultures, SALL1 knockdown significantly increased spheroid size in both Huh7 and Hep3B cells (Figure 2E,G). CCK8 assays confirmed higher proliferation in the SALL1-knockdown cells (Figure 2F,H). These findings suggest that reduced SALL1 expression promotes a more aggressive in vitro phenotype of well-differentiated HCC cell lines.

### 3.3. Loss of SALL1 Expression Drives Tumor Growth in a Xenograft Mouse Model

**Figure 3:**
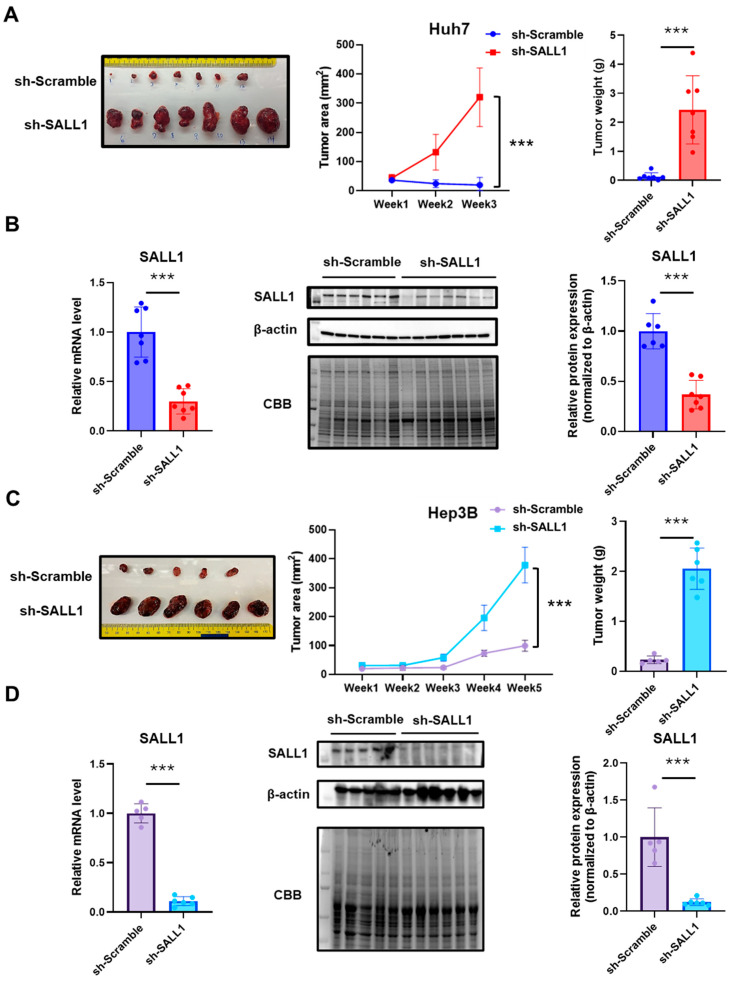
**Loss of SALL1 expression drives tumor growth in a CDX model.** (**A**,**C**) Xenograft mouse models using immunodeficient mice implanted with Huh7 (**A**) or Hep3B (**C**) cells expressing sh-Scramble or sh-SALL1. Representative images of excised tumors (left), tumor growth curves (middle), and final tumor weights at the experimental endpoint (right). (Huh7: sh-Scramble, n = 7; sh-SALL1, n = 7; Hep3B: sh-Scramble, n = 5; sh-SALL1, n = 6). (**B**,**D**) Detection of SALL1 knockdown in excised xenograft tumors. Expression analysis in tumors derived from Huh7 (**B**) and Hep3B (**D**) cells. RT-qPCR analysis of *SALL1* mRNA levels (left) and Western blotting of SALL1 protein levels (middle and right). Western blot band intensities of SALL1 were quantified and normalized to β-actin as an internal control. Data are presented as mean ± SEM. Statistical significance was determined using two-tailed Student’s *t*-test. *** *p* < 0.001 compared with sh-Scramble group (control).

SALL1-knockdown HCC cell lines that exhibited enhanced cell proliferation in vitro were transplanted into the right flank of immunodeficient (NSG) male mice to investigate whether they show a similar tendency of cell proliferation in the in vivo environment. Consistent with the in vitro results, tumor growth, size, and weight of the SALL1-knockdown cells were significantly increased compared with the control group expressing SALL1 in both Huh7 and Hep3B cells (Figure 3A,C). Similarly, in female mice, tumor growth, size and weight were significantly increased in SALL1-knockdown cells compared with the control group with both Huh7 and Hep3B cells (Supplementary Figure S2A,C). Comparable results were observed in both sexes. SALL1 expression in tumors was verified at the mRNA and protein levels (Figure 3B,D, Supplementary Figure S2B,D). To exclude the possibility that lentiviral infection itself suppressed cell proliferation, untreated Huh7 and Hep3B cells (NT) were also transplanted simultaneously. No significant difference in tumor growth rate was observed between untreated and control cells (Supplementary Figure S3A,B). These results demonstrate that SALL1 knockdown markedly promotes tumorigenesis and accelerates malignant progression in HCC cells. Taken together, these results suggest that SALL1 knockdown in Huh7 and Hep3B promote tumor growth and progression of HCC.

### 3.4. Identification of Novel Target Gene Candidates of SALL1

**Figure 4:**
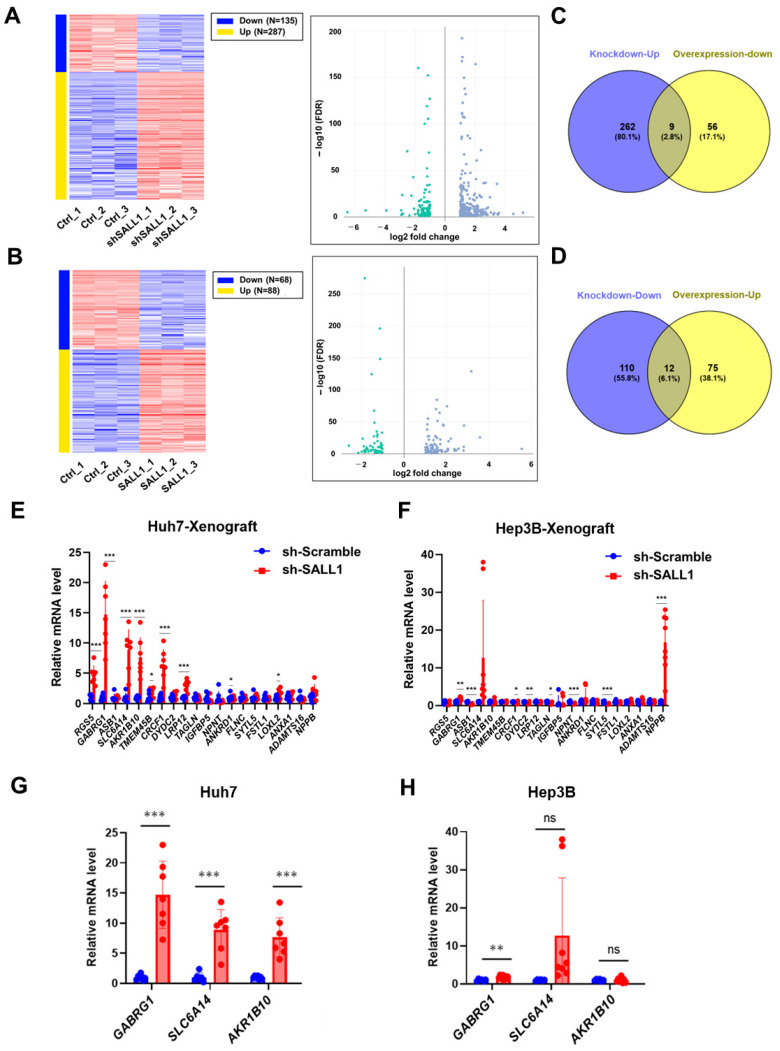
**Identification of novel SALL1 target genes.** (**A**,**B**) RNA-seq-based gene expression profiling in Huh7 cells. Differential expression analysis following SALL1 knockdown (**A**) or SALL1 overexpression (**B**). Heatmaps (left) and volcano plots (right) are shown. In the heatmap, red indicates upregulation, whereas blue indicates downregulation. The volcano plots highlight genes meeting the thresholds of FDR < 0.05 and mean fold change ≥ 2. Blue plots represent genes upregulated by SALL1 knockdown (**A**) or SALL1 overexpression (**B**), whereas green plots represent genes downregulated by SALL1 knockdown (**A**) or SALL1 overexpression (**B**). (**C**,**D**) Venn diagram analysis to identify candidate SALL1-regulated target genes based on reciprocal expression changes in the two RNA-seq datasets. (**C**) Genes significantly upregulated upon SALL1 knockdown and significantly downregulated upon SALL1 overexpression. (**D**) Genes significantly downregulated upon SALL1 knockdown and significantly upregulated upon SALL1 overexpression. Duplicated gene symbols in the differential expression analysis were collapsed into unique genes before Venn diagram analysis. (**E**,**F**) Expression analysis of candidate target genes identified by Venn diagram analysis using xenograft tumor samples. (**E**) Huh7-derived tumors. (**F**) Hep3B-derived tumors. (**G**,**H**) RT-qPCR validation of representative candidate target genes (*GABRG1*, *SLC6A14*, and *AKR1B10*) in SALL1-knockdown Huh7 (**G**) and sh-SALL1 Hep3B cells (**H**) cells. RNA-seq was performed using biological triplicates for each group (n = 3 per group). For RT-qPCR validation, Huh7 cells included sh-Scramble (n = 7) and sh-SALL1 (n = 7), whereas Hep3B cells included sh-Scramble (n = 6) and sh-SALL1 (n = 8). Data are presented as mean ± SEM. Statistical significance was determined using two-tailed Student’s *t*-test. * *p* < 0.05, ** *p* < 0.01, *** *p* < 0.001 compared with sh-Scramble group (control). ns, non-significant.

To identify SALL1 downstream target genes and investigate the mechanism by which SALL1 knockdown promotes tumor growth in Huh7 and Hep3B cells, RNA-seq was performed on SALL1-knockdown and SALL1-overexpressing Huh7 cells. Differential expression analysis revealed significant changes in levels of numerous mRNAs following SALL1 knockdown or overexpression. Notably, under the knockdown conditions employed, 135 genes (as reflected by relative mRNA levels) were significantly downregulated, whereas 287 genes were significantly upregulated (Figure 4A). Similarly, 68 genes were significantly downregulated and 88 genes were significantly upregulated by SALL1 overexpression (Figure 4B). Venn diagram analysis identified 21 genes, including SALL1, that reciprocally changed between the two conditions: nine genes were upregulated upon SALL1 knockdown and downregulated upon SALL1 overexpression, whereas 12 genes showed the opposite pattern (Figure 4C,D). Furthermore, direct mRNA quantification analysis of the 20 genes, excluding SALL1, in tumors derived from Huh7 and Hep3B cells (Figure 4E,F) showed that *GABRG1* and *SLC6A14* mRNAs were increased upon SALL1 knockdown in tumors derived from both Huh7 and Hep3B cells (Figure 4G,H). Furthermore, consistent with the results obtained from tumor samples (in vivo samples), these mRNAs were also significantly increased in cultured cells collected from dishes (in vitro samples) containing both Huh7 and Hep3B cells following SALL1 knockdown (Supplementary Figure S4A). Although *AKR1B10* mRNA tends to increase only in Huh7, both in tumor-derived samples and cultured cells (Figure 4G,H) (Supplementary Figure S4A), it was significantly increased in HCC compared with normal tissues in the TCGA dataset analysis. Furthermore, *SLC6A14* was significantly upregulated in HCC compared with normal tissues while *GABRG1* expression was not elevated in HCC (Supplementary Figure S4B). Thus, *SLC6A14* and *AKR1B10* were identified as candidate target genes. Although overexpression of *GABRG1* was not confirmed in HCC, its expression was altered by SALL1 knockdown in both tumors and cultured cells. Therefore, it was also identified as a candidate target gene.

### 3.5. SALL1 Binds to the Promoters of Candidate Target Genes

**Figure 5:**
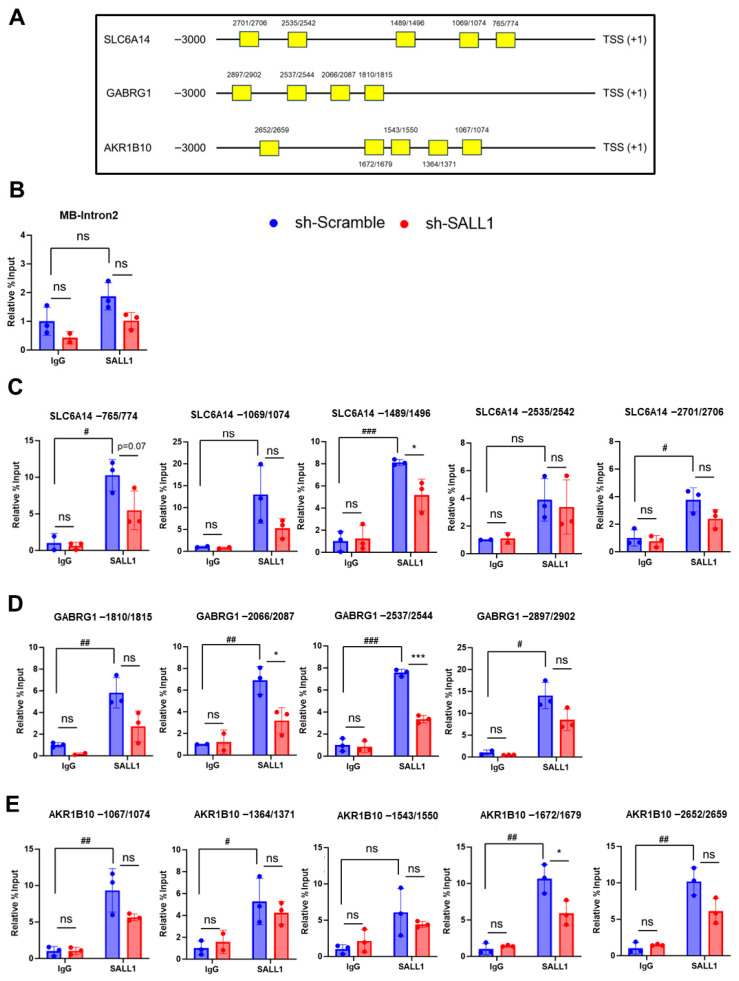
**Binding of SALL1 to the promoters of target genes.** ChIP-qPCR analysis of SALL1 enrichment at the predicted SALL1 binding region. (**A**) Schematic illustration of the predicted SALL1 binding sites in the promoter regions of candidate target genes. (**B**) ChIP-qPCR analysis of a negative control region. A muscle-specific genomic region that is not expected to be accessible to SALL1 in liver-derived cells was used as a negative control locus. (**C**–**E**) Predicted binding regions in *SLC6A14* (**C**), *GABRG1* (**D**), and *AKR1B10* (**E**) using IgG and anti-SALL1 immunoprecipitation. * *p* < 0.05, *** *p* < 0.001 compared with sh-Scramble group (control). All experiments were performed using three biological replicates for each group (n = 3 per group). Data are presented as mean ± SEM. Statistical significance was determined using two-tailed Student’s *t*-test. ^#^ *p* < 0.05, ^##^ *p* < 0.01, ^###^ *p* < 0.001 compared with IgG (IgG vs. SALL1 antibody) in sh-Scramble groups. ns, non-significant.

To determine whether SALL1 binds to the promoters of the three genes identified by the screening analysis, ChIP assays were performed using Huh7 cells. In this analysis, the genomic region spanning 3000 bp upstream of the transcription start site [TSS] of each candidate target gene was defined as the promoter region to be examined. Putative binding sites were identified by searching the 3000 bp region upstream of the TSS (+1) of each candidate target gene based on the SALL1 consensus sequence previously identified in the prior studies [40]. Within this upstream region, five putative SALL1 binding sites were predicted for *SLC6A14* and *AKR1B10*, whereas four sites were predicted for *GABRG1* (Figure 5A). These predicted regions were subsequently analyzed to assess SALL1 binding. ChIP-qPCR analysis showed enrichment of SALL1 at the putative promoter regions, indicating promoter occupancy/binding. As a negative control, a genomic region predicted not to be bound by SALL1 was used. Specifically, an intronic region of the muscle-specific *myoglobin* (*MB*) gene, which is not expected to be accessible to liver transcription factors, was used as a negative control. ChIP-qPCR with an anti-SALL1 antibody did not show significant enrichment at this region compared with IgG control immunoprecipitation, confirming the specificity of the assay (Figure 5B). For *SLC6A14*, among the five predicted regions, significant enrichment was detected at the region located −1489–1496 bp upstream of the TSS in ChIP assays using anti-SALL1 antibody, compared with IgG control immunoprecipitation (Figure 5C). Notably, this enrichment was significantly reduced upon SALL1 knockdown (Figure 5C). These results suggest that SALL1 binds to the −1489–1496 bp region of the *SLC6A14* promoter. The same analyses were performed for *GABRG1* and *AKR1B10*. For *GABRG1*, significant enrichment was observed at the regions located −2066–2087 bp and −2537–2544 bp upstream of the TSS in ChIP assay using an anti-SALL1 antibody, compared with IgG control immunoprecipitation, and this was significantly reduced upon SALL1 knockdown (Figure 5D). For *AKR1B10*, a similar trend was observed at the region located −1672–1679 bp of the TSS (Figure 5E). Collectively, these results suggest that SALL1 binds to the promoter regions of *SLC6A14*, *GABRG1*, and *AKR1B10*, and that these genes are potential direct SALL1 targets.

## 4. Discussion

This study revealed that SALL1 expression is reduced in HCC and this decrease in SALL1 leads to enhanced cell proliferation and accelerated tumor growth in HCC cells in vitro and in vivo. Downstream gene networks were further identified that are potentially regulated by SALL1. Collectively, these findings provide support for SALL1 as a tumor suppressor in HCC.

Expression analysis of SALL1 using database and human samples revealed that SALL1 expression is reduced in HCC and is associated with poor prognosis in humans. Based on these observations, the effect of reduced SALL1 expression in HCC was examined using cell culture and xenograft models. Functional analyses demonstrated that SALL1 knockdown in Huh7 and Hep3B cells promotes cell proliferation and tumor growth, indicating that loss of SALL1 confers a growth advantage to HCC cells. Taken together, these findings suggest that reduced SALL1 expression may contribute to HCC malignancy in the clinical setting, particularly by promoting tumor growth. A previous study showed that overexpression of SALL1 suppresses proliferation of HCC cell lines [34]. In contrast, the SALL1 knockdown experiments performed in the present study resulted in enhanced cell proliferation and tumor formation in both Huh7 and Hep3B cells. Collectively, these results further support and strengthen previous reports proposing SALL1 as a tumor suppressor in HCC [34]. Furthermore, *GABRG1*, *SLC6A14*, and *AKR1B10* were identified as novel SALL1 target genes. These results suggest that SALL1 may exert its tumor-suppressive function, at least in part, through transcriptional regulation of downstream target genes.

SALL1 was reported to function as a tumor suppressor in breast cancer and glioma, while acting as an oncogene in acute myeloid leukemia and colorectal cancer, highlighting its context-dependent and dual roles depending on tumor type and genetic background [29,32,33]. Although accumulating evidence has revealed diverse functions of SALL1 across multiple cancer types, the identification of downstream target genes regulated by SALL1 in cancer remains limited. Elucidating SALL1-regulated gene networks in cancer would therefore provide important insights into SALL1-centered cancer biology. In this study, three genes, *GABRG1*, *SLC6A14*, and *AKR1B10*, were identified as novel SALL1 target candidates. As these three genes were negatively regulated by SALL1, they may play an oncogenic role in HCC development. GABRG1 (gamma-aminobutyric acid receptor subunit gamma-1) encodes the γ1 subunit of the GABA_A receptor [41]. To date, reports describing a direct role for GABRG1 alone in cancer are limited, and no studies have established GABRG1 as an oncogenic driver or prognostic determinant for HCC. Although database analysis showed that GABRG1 expression is not increased in HCC, GABRG1 was consistently increased following SALL1 knockdown in both Huh7 and Hep3B cells. These findings suggest that GABRG1 may be associated with the phenotype driven by SALL1 knockdown and therefore was identified in this study as a candidate downstream SALL1 target. However, the biological significance of GABRG1 in HCC progression, if any, remains to be elucidated. SLC6A14 is a broad-spectrum amino acid transporter responsible for the uptake of most neutral and basic amino acids, excluding aspartate and glutamate. SLC6A14 was reported to indirectly activate the mTOR signaling pathway through increasing amino acid supply, thereby contributing to tumor growth [42]. AKR1B10 (aldo-keto reductase family member B10) encodes an enzyme involved in retinoid metabolism, catalyzing the conversion of retinaldehyde to retinol [43]. In the retinoid metabolic pathway, retinol is sequentially converted to retinaldehyde and ultimately to retinoic acid (RA), which activates RAR/RXR signaling to promote cell differentiation and suppress cell proliferation [44]. Increased AKR1B10 expression was shown to reduce RA production, leading to attenuation of RAR/RXR signaling and increased carcinogenic risk associated with impaired differentiation [45]. AKR1B10 is highly expressed in HCC tissues, and it was reported that AKR1B10 knockdown inhibits proliferation of HCC cells as well as xenografted HCC tumors [46]. Accordingly, among the three genes identified as downstream target candidates, *SLC6A14* and *AKR1B10* were reported to potentially contribute to malignant phenotypes, including enhanced proliferation and dedifferentiation. Upregulation of these genes may therefore be associated with the increased cell proliferation and tumor growth observed upon SALL1 knockdown. However, the individual contribution of each gene to the observed phenotypes remains to be determined. Thus, future studies using rescue and gene knockdown approaches will be required to identify downstream factors that are essential for mediating the phenotypic effects of SALL1 loss.

Although the present data do not establish a complete causal relationship at the mechanistic level, the consistent tumor-promoting phenotypes observed across multiple experimental models in this study (SALL1 knockdown, use of two different HCC cell lines) support the biological significance and potential clinical relevance of SALL1 downregulation in HCC. Thus, the restoration of SALL1 expression could suppress, at least in part, these malignant phenotypes, and may represent a potential therapeutic rationale for HCC. Future studies incorporating SALL1 re-expression (rescue) experiments, together with comprehensive identification of downstream pathways and stratification of responsive tumor subtypes or disease stages, would be informative to more rigorously assess the feasibility of targeting SALL1-related pathways.

Taken together, the present study evaluated the biological impact of SALL1 downregulation in HCC and provides evidence supporting the notion that SALL1 may function as a tumor suppressor in HCC. Furthermore, through RNA-seq and ChIP-qPCR analyses, we partially identified downstream targets of SALL1 and delineated a subset of the transcriptional regulatory network altered by SALL1 loss. Future studies incorporating rescue experiments and validation using clinical specimens would be valuable to further clarify causality and clinical relevance of these findings.

This study has several limitations. First, our loss-of-function analyses were performed using a single shRNA construct, and additional independent approaches would further strengthen the specificity of the findings. In addition, rescue assays involving re-expression of SALL1 after knockdown were not performed in this study, and therefore further validation would be helpful to confirm that the observed phenotypic changes were specifically attributable to SALL1 knockdown. However, SALL1 knockdown was consistently confirmed at both the mRNA and protein levels in two HCC cell lines, and similar phenotypic effects were observed across multiple in vitro and in vivo assays. Further studies incorporating additional knockdown approaches such as siRNA-mediated knockdown and rescue experiments will be useful to further confirm the causal contribution of SALL1 loss to the observed phenotypes. Second, although RNA-seq and ChIP analysis identified candidate downstream targets associated with SALL1 knockdown, the present study was not designed to determine the relative contribution of each target to the observed phenotypic changes. Further characterization of these candidate targets may help to refine our understanding of the mechanisms associated with reduced SALL1 expression in HCC.

## 5. Conclusions

The present findings suggest that SALL1 may function as a tumor suppressor in HCC. SALL1 expression was reduced in HCC tissues at both the mRNA and protein levels, and SALL1 knockdown promoted cell proliferation and tumor growth in HCC cell lines. These results indicate that downregulation of SALL1 may contribute to HCC progression. Although the precise mechanisms driving the decrease in SALL1 remains to be fully clarified, this study provides evidence supporting the possible involvement of SALL1 loss in the malignant progression of HCC. Further studies will be needed to better define the molecular pathways associated with SALL1 and to evaluate its potential as a therapeutic target in HCC.

## Data Availability

The public datasets analyzed in this study are available from the following online resources: UCSC Xena (https://xenabrowser.net/, accessed on 6 April 2026), UALCAN (https://ualcan.path.uab.edu/, accessed on 6 April 2026), and the Human Protein Atlas (https://www.proteinatlas.org/, accessed on 6 April 2026). These resources were used for in silico analyses of gene expression and survival data. No new public dataset was generated through these platforms, and all analyzed data are available from the respective website. The scRNA-seq dataset used in this study was GSE242889, which is publicly available through the Gene Expression Omnibus (GEO).

## Supplementary Materials

The following supporting information can be downloaded at https://www.mdpi.com/article/10.3390/cancers18091355/s1: Figure S1: Expression analysis of SALL1 in tumor grades and HCC cell lines; Figure S2: Xenograft mouse model using female immunodeficient mice; Figure S3: Xenograft mouse model including untreated cells; Figure S4: Expression analysis of novel candidate SALL1 target genes; Table S1: Patient information; Table S2: Primers used in mRNA quantification; Table S3: Sequence of shRNA; Table S4: Primers used in ChIP-qPCR.
