# Supplementary material for: Loss of SALL1 Promotes Hepatocellular Carcinoma Growth and Is Associated with Poor Clinical Outcome
Source: Cancers (Basel). 2026 Apr 24;18(9):1355. doi: 10.3390/cancers18091355 (PMC13162894; doi:10.3390/cancers18091355)
Supplement: Supplementary file 1 [file cancers-18-01355-s001.zip › cancers-4248488-supplementary-Author done.pdf]

# Supplementary figures and tables

Supplementary Figure 1 Page 2
Supplementary Figure 2 Page 3
Supplementary Figure 3 Page 5
Supplementary Figure 4 Page 6

Supplementary Table 1 Page 7
Supplementary Table 2 Page 8
Supplementary Table 3 Page 9
Supplementary Table 4 Page 9

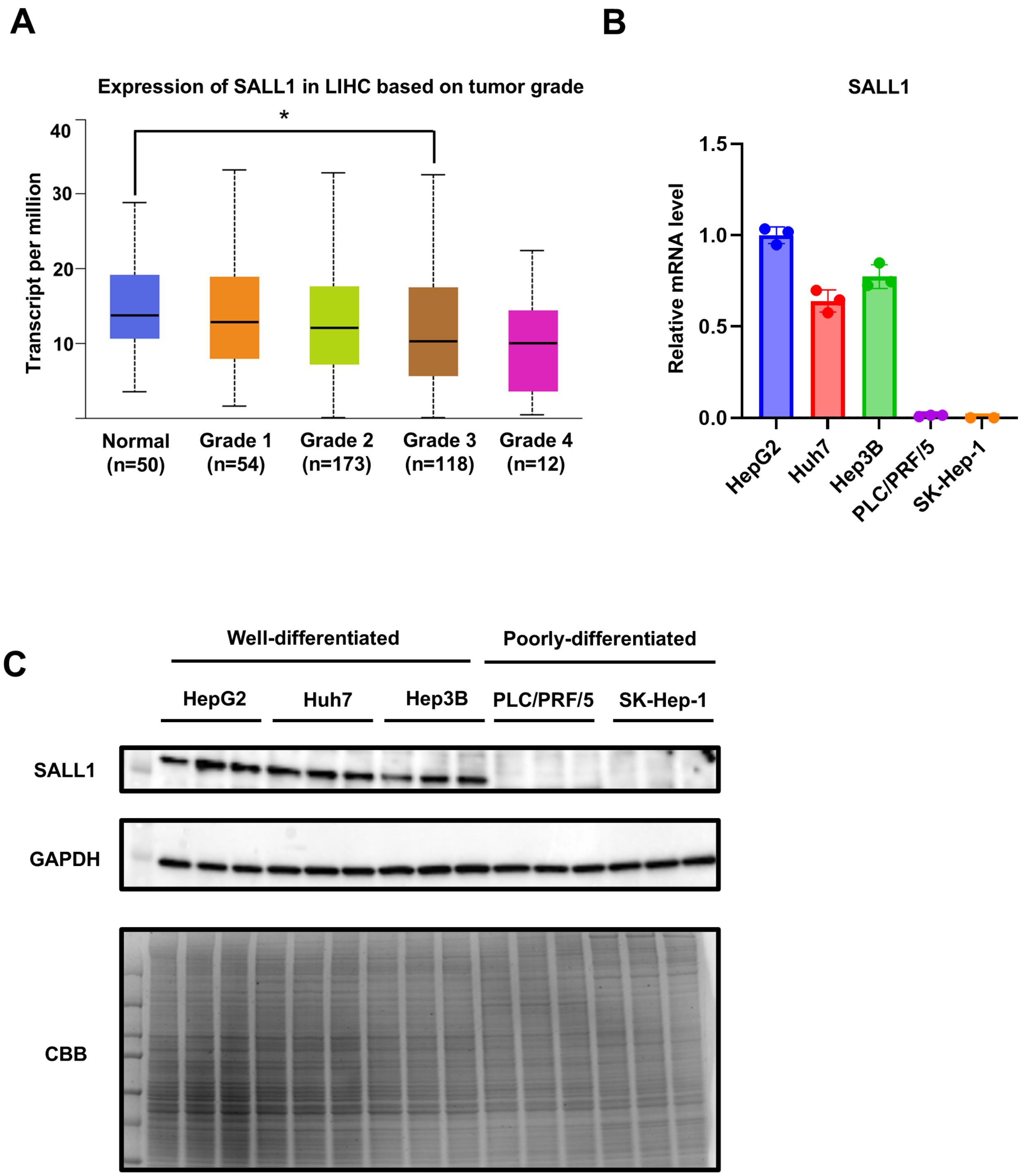


**Figure S1. Expression analysis of SALL1 in tumor grades and HCC cell lines**
**(A)** SALL1 expression across tumor histological grades (grades 1-4). **(B, C)** Comparison of SALL1 expression across five HCC cell lines. **(B)** RT-qPCR analysis of *SALL1* mRNA levels. **(C)** Western blot analysis of SALL1 protein levels. **P*<0.05 compared with Normal.

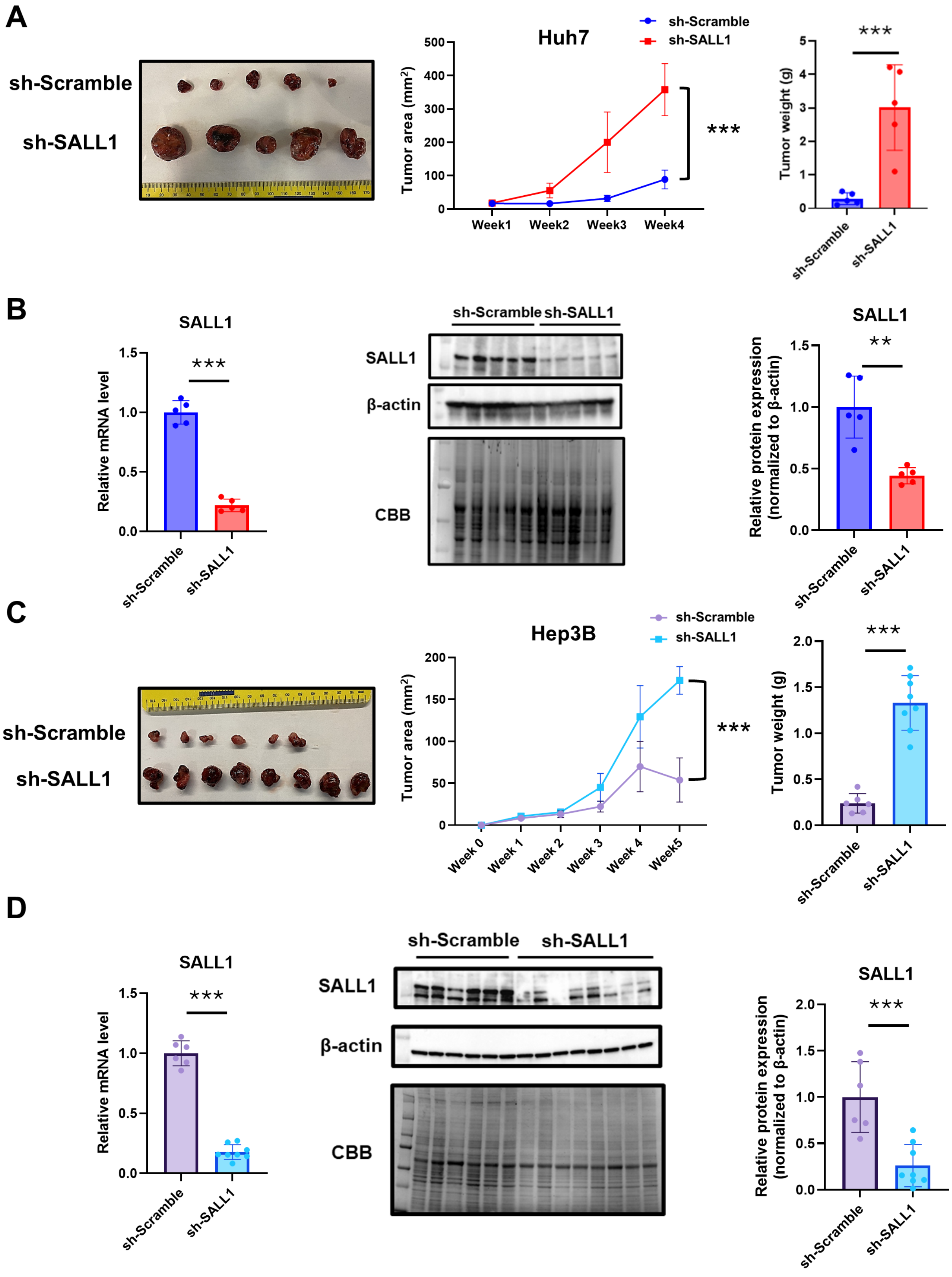

A
sh-Scramble
sh-SALL1
Huh7
sh-Scramble
sh-SALL1
Tumor area (mm²)
500
400
300
200
100
0
Week1
Week2
Week3
Week4
***
Tumor weight (g)
5
4
3
2
1
0
sh-Scramble
sh-SALL1
***
B
SALL1
Relative mRNA level
1.5
1.0
0.5
0.0
***
sh-Scramble
sh-SALL1
sh-Scramble
sh-SALL1
SALL1
β-actin
CBB
SALL1
Relative protein expression
(normalized to β-actin)
1.5
1.0
0.5
0.0
**
sh-Scramble
sh-SALL1
C
sh-Scramble
sh-SALL1
Hep3B
sh-Scramble
sh-SALL1
Tumor area (mm²)
200
150
100
50
0
Week 0
Week 1
Week 2
Week 3
Week 4
Week5
***
Tumor weight (g)
2.0
1.5
1.0
0.5
0.0
***
sh-Scramble
sh-SALL1
D
SALL1
Relative mRNA level
1.5
1.0
0.5
0.0
***
sh-Scramble
sh-SALL1
sh-Scramble
sh-SALL1
SALL1
β-actin
CBB
SALL1
Relative protein expression
(normalized to β-actin)
2.0
1.5
1.0
0.5
0.0
***
sh-Scramble
sh-SALL1

**Figure S2. Xenograft mouse model using female immunodeficient mice**
**(A, C)** Xenograft mouse models using immunodeficient mice (female) implanted with Huh7 **(A)** or Hep3B **(C)** cells. Images of excised tumors (left), tumor growth curves (middle), and tumor weights (right). **(B, D)** Detection of SALL1 knockdown in excised xenograft tumors. Expression analysis in tumors derived from Huh7 **(B)** and Hep3B **(D)** cells. RT-qPCR analysis of *SALL1* mRNA levels (left) and Western blotting of SALL1 protein levels (middle and right). Westen blot band intensities of SALL1 were quantified and normalized to β-actin as an internal control. **$P$<0.01, ***$P$ < 0.001 compared with sh-Scramble group (Control).

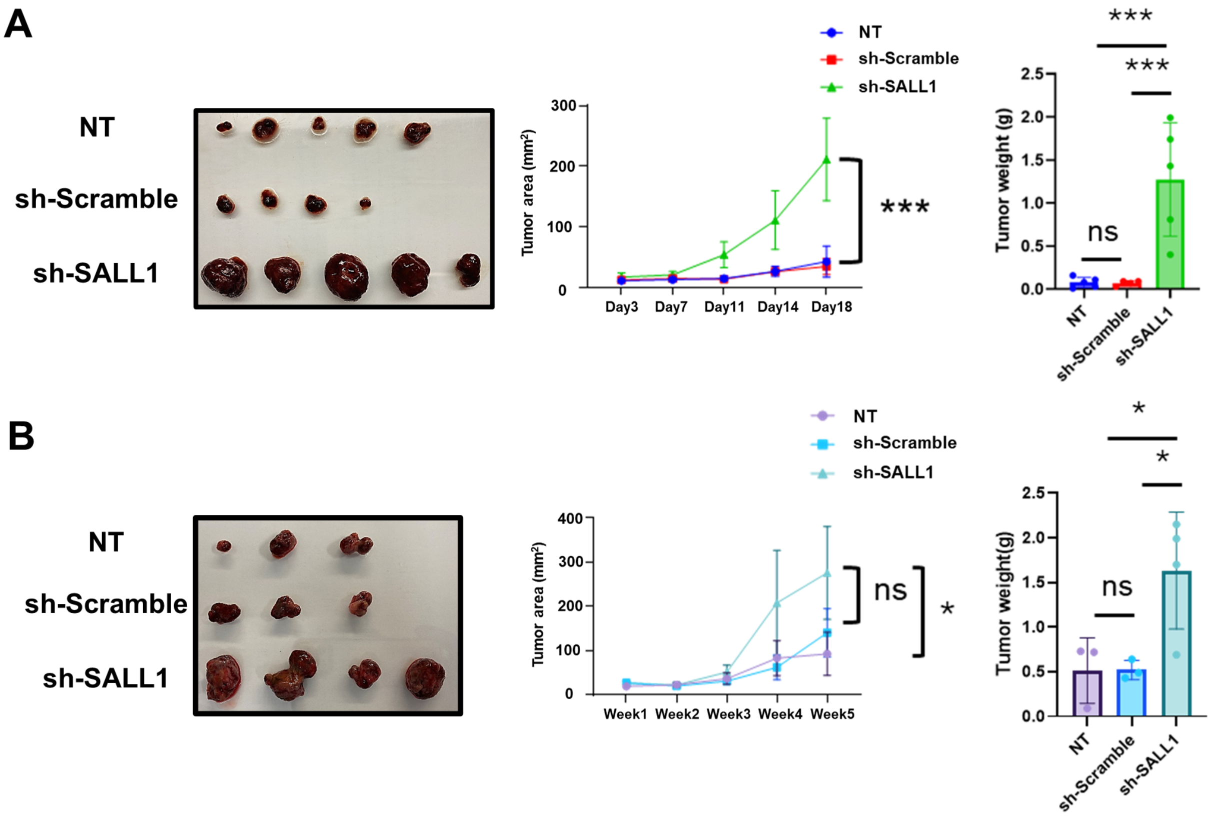


**Figure S3. Xenograft mouse model including untreated cells**

Xenograft mouse model including untreated (NT) cells. **(A)** Results from mice implanted with Huh7 cells and **(B)** results from mice implanted with Hep3B cells. Images of excised tumors (left), tumor growth curves (middle), and tumor weights (right). **P* < 0.05, ****P* < 0.001 compared with untreated (NT) group or sh-Scramble group.

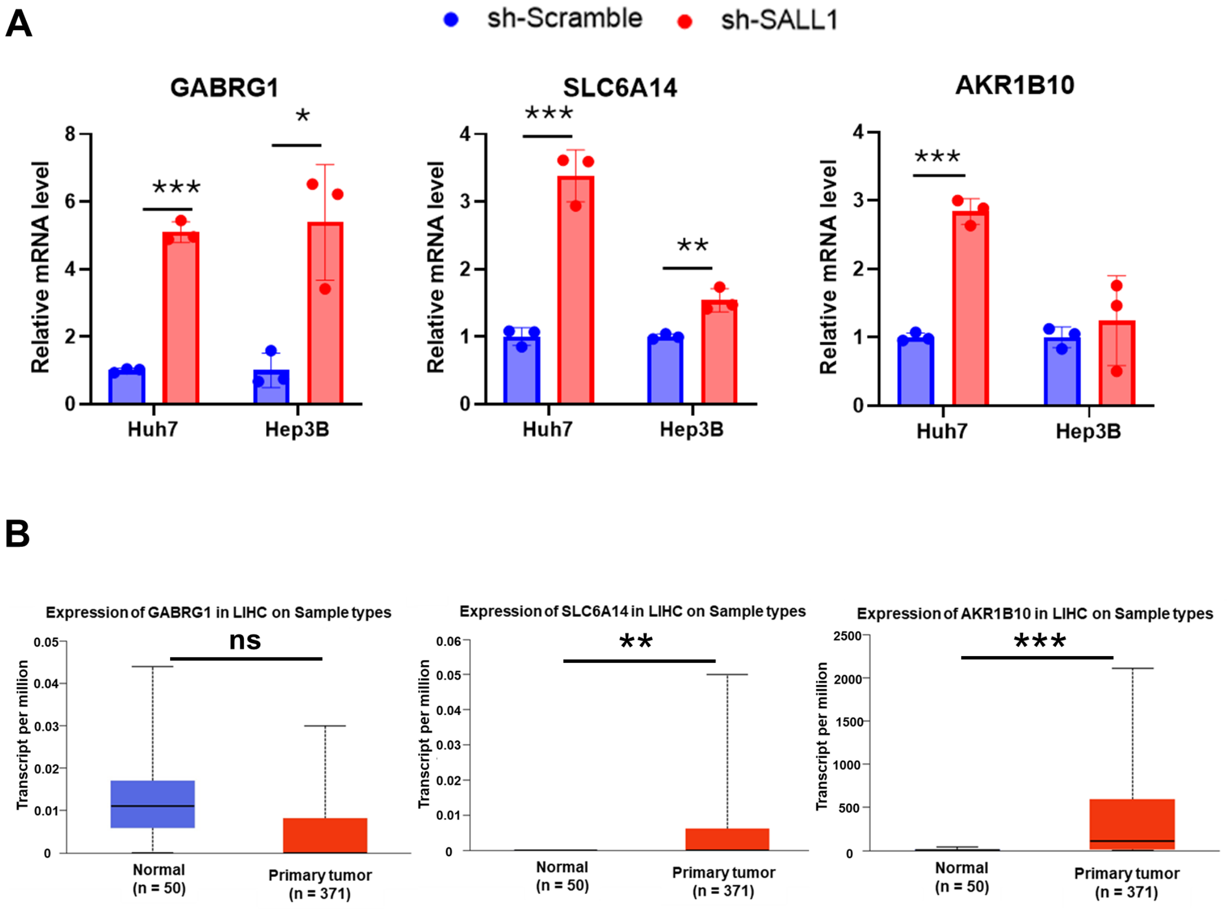


**Figure S4. Expression analysis of novel candidate SALL1 target genes**
**(A)** RT-qPCR analysis of candidate SALL1 target gene expression in SALL1-knockdown cells. **(B)** Expression analysis of candidate target genes in HCC based on the TCGA database. *$P$< 0.05, **$P$ < 0.01, ***$P$ < 0.001 compared with sh-Scramble group (Control) or normal.

**Supplementary Table 1. Patient information**

| Category | Characteristic | Value |
|---|---|---|
| Clinical characteristics | Total case | 19 |
| | Age: Average (Range) | 62.1 (23-78) |
| | Gender: n (%) | Male: 14 (73.7), Female: 5 (26.3) |
| | Virus | 0 |
| | Alcohol: n (%) | 1 (5.2) |
| | Fatty liver: n (%) | 6 (31.5) |
| | NASH: n (%) | 1 (5.2) |
| | Diabetes: n (%) | 2 (10.5) |
| | Hyperlipidemia: n (%) | 2 (10.5) |
| | Cirrhosis: n (%) | 2 (10.5) |
| Pathology | Well-differentiated: n (%) | 1 (5.2) |
| | Well to moderately differentiated: n (%) | 2 (10.5) |
| | Moderately differentiated: n (%) | 5 (26.3) |
| | Others: n (%) | 11 (57.9) |
| Primary Clinical Diagnosis | Space-occupying lesion of the liver: n (%) | 12 (63.1) |
| | Secondary malignant neoplasm of the liver: n (%) | 1 (5.2) |
| | HCC: n (%) | 1 (5.2) |
| | Malignant neoplasm of the liver: n (%) | 4 (21.0) |
| | Liver mass: n (%) | 1 (5.2) |
| Etiology | Metabolic liver disease: n (%) | 10 (52.6) |
| | Immune-mediated liver disease: n (%) | 9 (47.3) |

**Supplementary Table 2. Primers used in mRNA quantification**

| Gene | Forward sequence (5′→3′) | Reverse sequence (5′→3′) |
|---|---|---|
| PPIA | CCCACCGTGTTCTTCGACATT | GGACCCGTATGCTTTAGGATGA |
| TBP | TTCGGAGAGTTCTGGGATTG | GGATTATATTCGGCGTTTCG |
| GAPDH | ATGACCCCTTCATTGACC | GAAGATGGTGATGGGATTTC |
| SALL1 | CCTGCAGATTCACGAGAGAAC | GTGCAGGGGTGCTATTCC |
| ADAMTS16 | TGCGATCACACAGAAACTCC | TGAAGGAAGTGGCCTTAGGA |
| AKR1B10 | GGAAAAGCAACGTTCTTGGA | GCTTCTCGATCTGGAAGTGG |
| ANKRD1 | TGAAGGCTGCTCTGGAGAAT | TCCTTCCAAGCATGCTCTATG |
| ANXA1 | TAAGCGAAACAATGCACAGC | GGTGACCTGTAAGGGCTTTCT |
| ASB4 | GTGGACACACAGCTTTGCAC | GCGTCTCCTCATCTTGGTTG |
| CRLF1 | GCTGGATATCCTGGATGTGG | CGGATCTGGTATTTGGCTTG |
| DYDC2 | GCCTCAAGGAAATGGAAATG | TGGTCTTCTTCGTGGAAACA |
| FLNC | AGTCCCTTCCCTGTCCATGT | CACCTTGAAGTCAGCCACCT |
| FSTL1 | CCAGACCAGGAGAACAACAAG | GGGTTGAGGCACTTGAGAAA |
| GABRG1 | AGTGCCTGCAAGAACATCGT | AGAGATCCATCGCAGTCACA |
| IGFBP5 | TTTGCCTCAACGAAAAGAGC | CGGAAGATCTTGGGGGAGTA |
| LOXL2 | CCTGGGGAGAGGACATACAA | CCATGGAGAATGGCCAGTAG |
| LRP12 | CTGGTTCATAAGGGCAAACC | CCAAATTGCACCTTCTGGAT |
| NPNT | AGGACAAATACGGTGCCAGT | TGCCTAAATCTAGGGCAGGA |
| NPPB | CTTCTTGCATCTGGCTTTCC | AGTTTGCCCTGCAAATGGT |
| RGS16 | AGGGCACACCAGATCTTTGA | GTCTGCAGGTTCATCCTCGT |
| SLC6A14 | ACTCTGGAGGGTGCTTCAAA | GAGTGGCAGCATCTTTCCAT |
| SYTL5 | TGGAAGTGCACTGTCTGTGA | GACAACATCAGTGCCGAGAA |
| TAGLN | AGGTGGCTCAGTTCCTGAAG | GCACTGCTGCCATGTCTTT |
| TMEM45B | CTGGAACTTTTCCGAACCAG | AAGGTGGGAACAGCACAAAC |

**Supplementary Table 3. Sequence of shRNA**

| shRNA | Sequence（5′→3′） |
|---|---|
| sh-Scramble | CCTAAGGTTAAGTCGCCCTCGCTCGAGCGAGGGCGACTTAACCTTAGG |
| sh-SALL1 | ACCCTTTGCTTGCACTATTTGCTCGAGCAAATAGTGCAAGCAAAGGGT |

**Supplementary Table 4. Primers used in ChIP-qPCR**

| Gene | Forward sequence (5′→3′) | Reverse sequence (5′→3′) | Region |
|---|---|---|---|
| GABRG1-1 | TCAGTGAGTTAGTGTGTGCATTC | AGTCAAGGTAGCCCATATCAAA | 1464-1469 |
| GABRG1-2 | CCCAAGGAGATACCCTTCAC | CTGCATGCAACAGTAGCTGA | 1810-1815 |
| GABRG1-3 | GGATCAGTTTCCCATAAAACAC | CCCGTATTCTCCAAAATCCA | 2066-2087 |
| GABRG1-4 | CCTTTACCCCCAGATTGTGA | CGTTTTCAAGTGGAGGCAAC | 2537-2544 |
| GABRG1-5 | TAATGGCGAAGCCCATGAAT | CCCCCAAAATTTGTTCAGG | 2897-2902 |
| SLC6A14-1 | CTGTTACCTGGGCAGACTGA | GAGGAAACATCCCAGCTTGA | 765-774 |
| SLC6A14-2 | CATGCACATCAGCCTTTGTT | GGGAATCTTGGCAGGTAAGG | 1069-1074 |
| SLC6A14-3 | GTGTTTGCTTTCCCTTCCAC | TGAAGAATTGCTCGAGACTAGG | 1489-1496 |
| SLC6A14-4 | ACCAGGTGTCCTTCCCTTTT | CCTGATGCAGAGTAGGCACT | 2400-2407,2432-2437 |
| SLC6A14-5 | CCCTCTCTCTTTCACCCATTC | CTCAAAGGCTCAAGAGATCCA | 2535-2542 |
| SLC6A14-6 | GCCTAGATTGCAAGGCAGAT | GGTGTGGGAAAACAGGAGAA | 2701-2706 |
| AKR1B10-1 | TGGGATTACAGGTGTGAGACA | GGCTTATGACAGCTCATAGAAGG | 1067-1074 |
| AKR1B10-2 | GGCCACCAGATTATGAGGAA | AGGTGGAGAATGCAGTGAGC | 1364-1371 |
| AKR1B10-3 | TGCAGTGCTCTATTCCCAGA | TGGATGACATGCAGTGCTA | 1543-1550 |
| AKR1B10-4 | GCGTGTCCAACCTCAGACTA | GGAATATGCATGCCACAGTAAA | 1672-1679 |
| AKR1B10-5 | AGCGGCACTACTCGTTCATT | CCTGGTTTTGTACCCTTAAGAA | 2652-2659 |
