# Supplementary figures and images for: Loss of SALL1 Promotes Hepatocellular Carcinoma Growth and Is Associated with Poor Clinical Outcome
Source: Cancers (Basel). 2026 Apr 24;18(9):1355. doi: 10.3390/cancers18091355 (PMC13162894; doi:10.3390/cancers18091355)

Figure.1

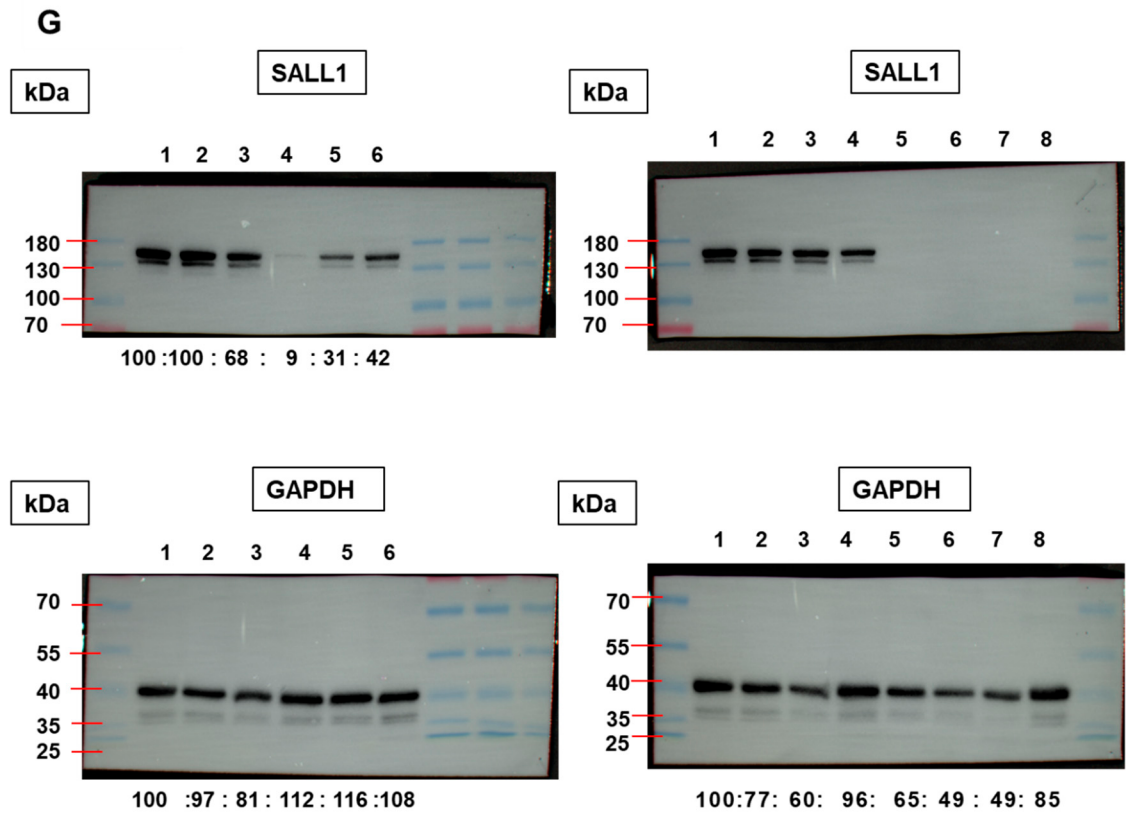

Figure.2

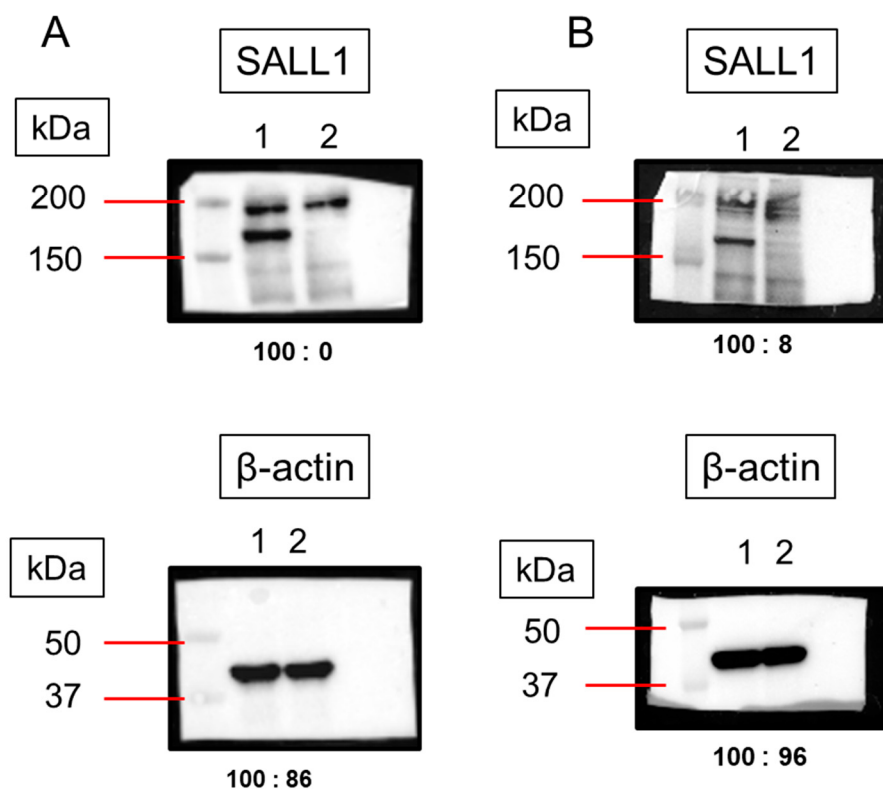

Figure.3

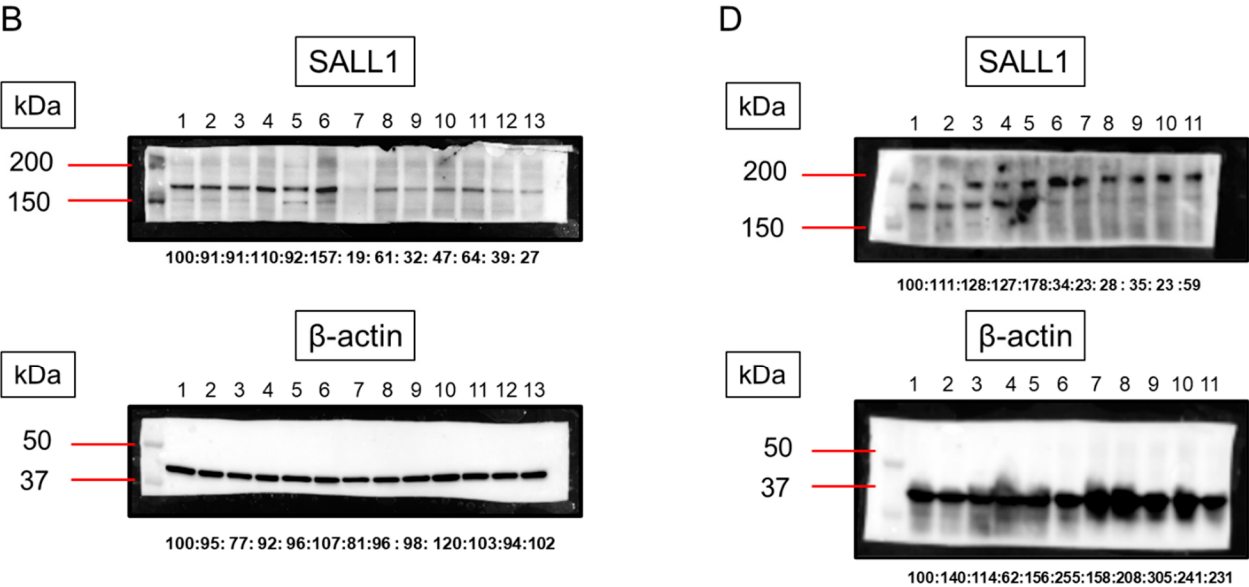

Sup Figure.1

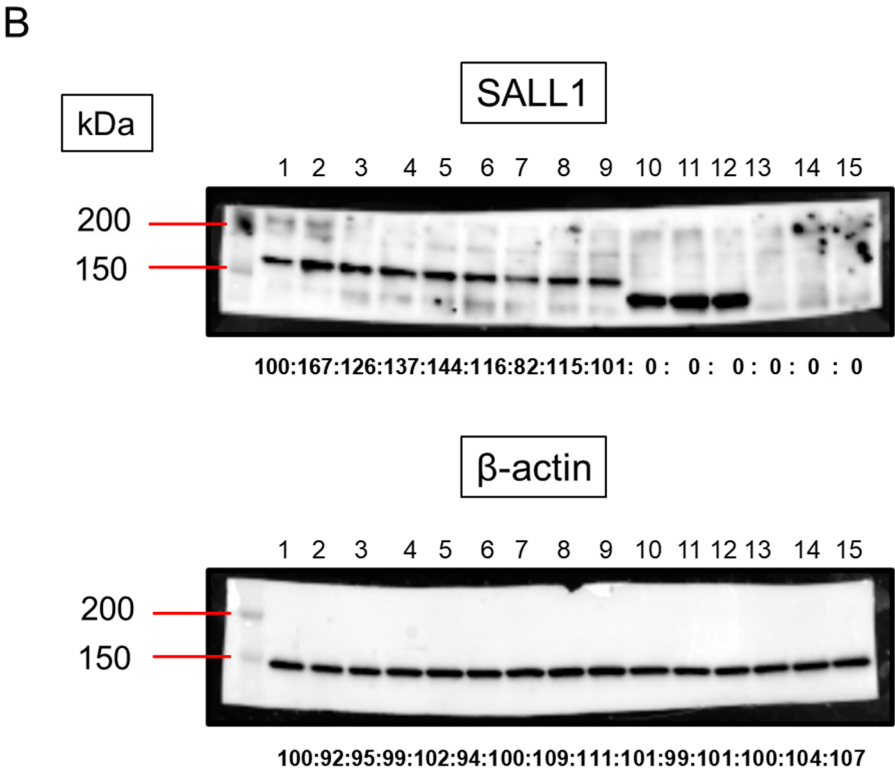

Sup Figure. 2

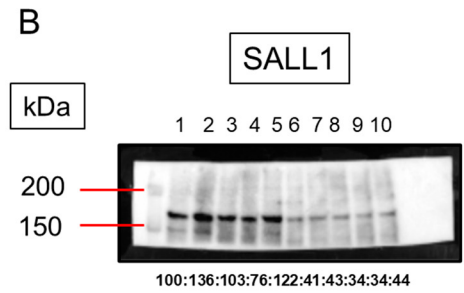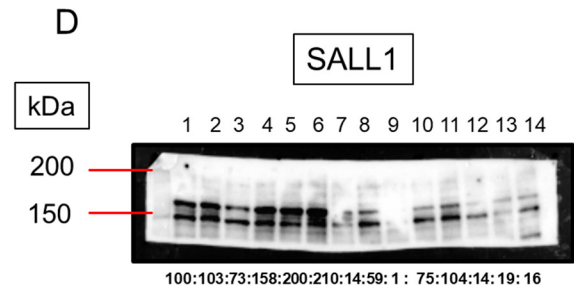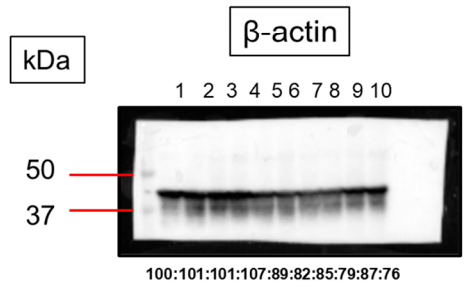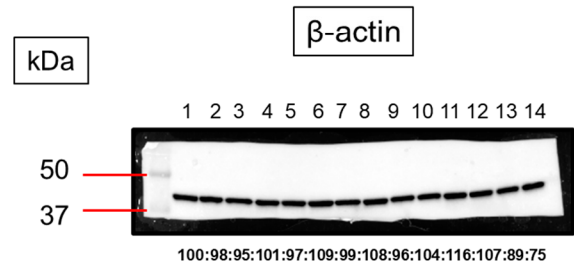

Supplement: Supplementary file 1 [file cancers-18-01355-s001.zip › cancers-4248488-WB raw image-updated.pdf]
